# Placebo and Nocebo Effects in Patients With Takotsubo Cardiomyopathy and Heart-Healthy Controls

**DOI:** 10.3389/fpsyt.2019.00549

**Published:** 2019-08-02

**Authors:** Elisabeth Olliges, Simon Schneider, Georg Schmidt, Daniel Sinnecker, Alexander Müller, Christof Burgdorf, Siegmund Braun, Stefan Holdenrieder, Hansjörg Ebell, Karl-Heinz Ladwig, Karin Meissner, Joram Ronel

**Affiliations:** ^1^Institute of Medical Psychology, Medical Faculty, LMU Munich, Munich, Germany; ^2^Medizinische Klinik und Poliklinik I, Klinikum rechts der Isar, Technische Universitaet Munich, Munich, Germany; ^3^German Centre for Cardiovascular Research (DZHK), partner site Munich Heart Alliance, Munich, Germany; ^4^Klinik für Herz- und Kreislauferkrankungen, Deutsches Herzzentrum Munich, Technische Universitaet Munich, Munich, Germany; ^5^Department of Cardiology, Heart and Vascular Centre Bad Bevensen, Bad Bevensen, Germany; ^6^Institute of Laboratory Medicine, Deutsches Herzzentrum Munich, Technische Universitaet Munich, Munich, Germany; ^7^Private Practitioner, Munich, Germany; ^8^Department of Psychosomatic Medicine and Psychotherapy, Klinikum rechts der Isar, Technische Universitaet Munich, Munich, Germany; ^9^Department of Epidemiology II, Helmholtz Zentrum, Munich, Germany; ^10^Division of Health Promotion, Coburg University of Applied Sciences, Coburg, Germany; ^11^Department of Psychosomatic Medicine, Klinik Barmelweid AG, Barmelweid, Switzerland

**Keywords:** placebo effects, nocebo effects, takotsubo cardiomyopathy, cardiological response, sympathetic nervous system

## Abstract

The etiology of takotsubo cardiomyopathy (TTC)—a rare, reversible, and acquired form of cardiac diseases—is not yet fully explained. An exaggerated activation of the sympathetic-nervous-system (SNS) following stressful psychosocial life events is discussed to be of key importance. In this experimental study, we tested whether TTC patients, compared to heart-healthy controls, respond more strongly to supporting placebo interventions and stressful nocebo interventions targeting cardiac function. In a single experimental session, 20 female TTC patients and 20 age matched (mean age 61.5 years, ± 12.89) catheter-confirmed heart-healthy women were examined. Saline solution was administered three times i.v. to all participants, with the verbal suggestion they receive an inert substance with no effects on the heart (neutral condition), a drug that would support cardiac functions (positive condition), and a drug that would burden the heart (negative condition). Systolic and diastolic blood pressure (DBP/SBP), heart rate (HR), endocrine markers cortisol (µg/dl), copeptin (pmol/l), and subjective stress ratings (SUD) were assessed to examine alterations of the SNS and the hypothalamic–pituitary–adrenal axis (HPA). Before and after each intervention SUD was rated. One pre and three post serum cortisol and copeptin samples were assessed, and a long-term electrocardiogram as well as non-invasive, continuous blood pressure was recorded. The study design elucidated a significant increase of SUD levels as a response to the nocebo intervention, while perceived stress remained unaffected during the preceding neutral and positive interventions. Increasing SUD levels were accompanied by higher SBP and an anticipatory increase of HR shortly prior to the nocebo intervention. SBP increased also as a response to positive verbal suggestions (Bonferroni-corrected p-values > .05). Alterations of cortisol and copeptin due to the interventions and significant placebo effects failed to appear. Interestingly no differences between TCC patients and controls could be found.These findings do not support the assumption of an exaggerated activation of the SNS as a discriminatory factor for TTC. Since especially the nocebo intervention revealed negative subjective and objective effects, our results underscore the urgent need to consider carefully the impact of verbal suggestions in the interaction with cardiac patients in daily clinical routine. This study is registered at the Deutsches Register Klinischer Studien (DRKS00009296).

## Introduction

Placebo effects are conceptualized as neurobiological phenomena, resulting from the positive psychosocial context, a treatment is embedded in. Correspondingly, a negative psychosocial context may induce negative clinical outcomes, referred to as “nocebo effects.” The current state of research suggests that placebo and nocebo effects are mediated by explicit expectations and shaped by different means; social observational learning (1), classical conditioning (2), and verbal suggestions (3). The doctor’s verbal suggestions inducing positive or negative outcome expectations are an important feature for placebo and nocebo effects (4–7). Placebo effects on functions linked to the central nervous system (CNS) such as pain or Parkinson’s disease have been extensively investigated and their mechanisms are well understood (6, 8). For example, placebo analgesia is often associated with the release of endogenous opioids, whereas placebo-induced motor improvement in patients with Parkinson’s disease could be connected to the release of dopamine in the dorsal striatum (8, 9). Within several studies, it has been demonstrated that placebo interventions can also affect peripheral organ functions (e.g., pulmonary and cardiovascular functions) controlled by the autonomic nervous system (ANS) (10–13), but results in this neglected area of placebo research are often ambiguous. For example, significant effects of verbal suggestions specifically targeting the diameter of coronary arteries could be observed during a coronary angiography. Here participants received intracoronary saline injections, together with the verbal suggestion the “drug” would widen the heart vessels and improve cardiac perfusion. Interestingly, the verbal suggestion led to coronary vasoconstriction accompanied by chest pain reduction. Acute psychological burden, HR and BP did not change significantly. Authors concluded that the coronary vasoconstriction was not caused by increased stress levels but by a reduction of sympathetic outflow and/or increase of parasympathetic outflow to the cardiac vessels (12).

Takotsubo cardiomyopathy (TTC) (also referred to as “stress-induced cardiomyopathy” or “broken heart syndrome”) is considered a very rare, reversible, and acquired form of primary myocardial disorders (14–16). TTC is characterized by an acute, functional disturbance in the contraction of the myocardium, primarily affecting mid and apical areas of the left ventricle, accompanied by symptoms and signs rather similar to those of the acute phase of a myocardial infarction (MI) (e.g., chest pain, dyspnea or alterations in the electrocardiogram or cardiac markers such as troponin), while the coronary arteries are mostly unaffected in TTC patients (17). Medeiros and colleagues found a similar impairment of systolic and diastolic function in TTCs and post MI patients, despite of their completely different pathophysiology (18). An increased sympathetic tone as well as a concomitant enhanced myocyte and microvascular catecholamine sensitivity is considered to increase the individual’s vulnerability and may therefore serve as a risk factor for the development of TTC (19).

Approximately 0.07–2.3% of patients, suspected with an acute coronary syndrome (ACS), are diagnosed with TTC after cardiac catheter examination, with almost 90% being postmenopausal women (14, 20–24). The etiology of TTC is not yet fully explained. A dysfunctional presentation and processing of external physiological or psychosocial stressors are assumed to initiate an inadequate activation of the sympathetic nervous system, and therefore a pathophysiological cascade of the TTC-patient’s myocardium (23, 25, 26). Triggers are not necessarily negative. A very small percentage of TTC patients (approximately 4%) experience a positive life event (e.g. a birthday party or the child’s wedding), prior to the onset of the disease. It is supposed that, positive as well as negative events are proceeding through analogous signal pathways in the central nervous system (26, 27).

Further, data on the recurrence of TTC varies, but relapses are not infrequent with approximately 1.5% to 2.4% per patient-year and a rate of 5% to 11.4% within the first 4 years (25, 28–30). Simultaneously, several studies found a significantly higher mortality rate in TTCs in comparison with a control group of the same age and sex (25, 31, 32). Apart from cardiovascular events, this appears to be due to an increased prevalence of non-cardiac comorbidities, which suggests a persistent pathology, presumably referring to an alteration of the sympathetic system, inherent in TTC patients (28, 33–36).

Based on these considerations, we investigated whether the cardiac regulation of TTC patients reacts more sensitively to positive and negative external stimuli than that of heart-healthy individuals. In a case–control study, we examined the cardiovascular response to placebo and nocebo interventions targeting the cardiac functions in 20 TTC patients on average two years after disease onset and 20 matched heart-healthy individuals. We hypothesized that in TTC patients cardiovascular and perceived stress parameters would be stronger regulated as a response to placebo and nocebo interventions compared to healthy individuals.

## Material and Methods

### Sample

This case–control study (controlled for age) included 20 women, diagnosed with TTC, and 20 volunteers (CG) free of significant coronary artery disease (vessel stenosis ≤30%, confirmed via heart catheterization in the past) (see **Table 1**). TTC patients were diagnosed regarding Mayo Clinic’s diagnostic criteria for Takotsubo Cardiomyopathy. These are: 1) transient hypokinesis, akinesis, or dyskinesis of the left ventricular mid segments with or without apical involvement; regional wall motion abnormalities extending beyond a single epicardial vascular distribution, with a stressful trigger often, but not always present, 2) absence of obstructive coronary disease or angiographic evidence of acute plaque rupture, 3) new electrocardiographic abnormalities (either ST-segment elevation and/or T-wave inversion) or modest elevation in cardiac troponin, 4) absence of a pheochromocytoma or myocarditis (37). Participants with significantly decreased ejection fraction (<55%) or low German proficiency, were excluded from the study. The mean time interval between the episode of TTC and the participation in the study was 24.61 months (±22.8). A total of 40 eligible women diagnosed at “Deutsches Herzzentrum” and “Medizinische Klinik und Poliklinik I, Klinikum rechts der Isar,” Technical University, Munich, were enrolled in the study and contacted *via* mail and followed-up by a phone call. The study protocol was approved by the institutional review board. All participants received 50 € compensation, borne by the Deutsches Herzzentrum, Munich.

**Table 1:** Baseline characteristics.

Characteristic	TTC	Controls	*p*
Age (years), *Mean (SD)*	61.65 (14.1)	61.35 (11.67)	.94^†^
Time point of examination *(n)*			.43^††^
09:00 am	5	8	
11:00 am	11	7	
01:00 pm	4	5	
Living in a relationship *(n)*	18	16	.64^††^
Number of children, *Median (IQR)*	2 (1–2)	1.5 (1–2)	.94^†^
Living condition *(n)*			
Alone	5	7	.73^††^
With partner and/or children	13	11	.51^††^
With children	4	7	.48^††^
Employment situation *(n)*			.64^††^
Fulltime	6	5	
Part time > 50%	2	5	
Part time < 50%	0	1	
Unemployed	1	0	
Retired	8	7	
Full time household	2	2	
Short Form Health Survey (SF-12)			
Physical component summary score (PCS)	55.8 (19.8)	50.3 (19.2)	.43^†^
Mental health component summary score (MCS)	56.2 (20.2)	51.3 (19.3)	.45^†^
Time since diagnosis (months), *Mean (SD)*	24.61 (22.8)		

Values are mean *±* SD or n (%). ^†^Mann–Whitney–U test, ^††^Chi-square-Test.

### Endpoints

The following parameter were chosen as primary endpoints in order to indicate alterations of the SNS and the HPA, the main peripheral pathways of the human stress system: Non-invasive continuous systolic (SBP) and diastolic blood pressure (DBP) as well as heart rate (HR) measured with Finapress Nova device (Finapres Medical Systems B.V.), as established indicators for the adaptive response to altered environmental, bio-psycho-social stimuli. Both cardiac functions are self-modifiable to attune the delivery of oxygenated blood by augmenting the beating frequency, respectively the pressure, with which the blood is pumped through the arteries (38). In addition, perceived stress was assessed by the “subjective units of distress scale” (SUD), an 11-point numeric rating scale from 0 (no stress) to 10 (maximal stress). Furthermore, blood samples were taken to measure cortisol (µg/dl) and copeptin (pmol/l). Cortisol has been shown to be proportionate to the degree of stress on a peripheral level. To gain a more direct insight in the stress level on the cerebral level, copeptin was chosen as a second humoral stress marker. Copeptin, a pre-hormone of vasopressin, is considered a relevant marker for acute, endogenous stress, especially associated with cardiological diseases (e.g. myocardial infarctions) (39–42).

### Procedure

**Figure 1:**
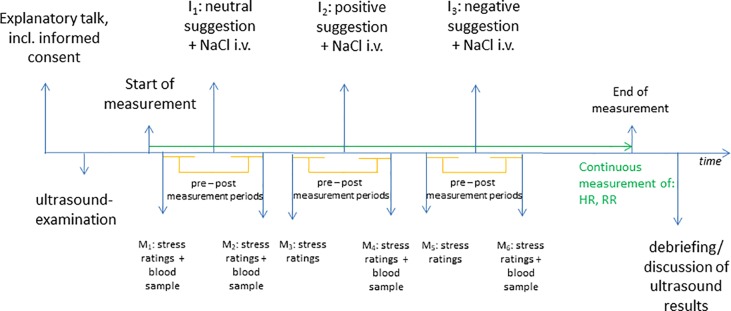
Procedure. M, Measurement; I, Intervention; HR, heart rate; RR, blood pressure.

The experiment was performed in the Department of Cardiology at Klinikum rechts der Isar, between 10:00 am and 1:00 pm in a cardiological outpatient lab. Participants were examined at different time points with no contact to each other; therefore an exchange of experiences during the experiment was not possible and no “placebo-by-proxy” effects could emerge (43). After obtaining informed consent, participants received a transthoracic echocardiography to assess standard parameters [e.g., septum thickness (mm) and ejection fraction (%)]. Thereafter, the study coordinator connected the participants to the Finapress Nova device (Finapres Medical Systems B.V.) and activated the continuous measurement of cardiovascular parameters [blood pressure (mmHg), heart rate (bpm)] while the attending physician established vascular access and took the first blood sample [cortisol (µg/dl) and copeptin (pmol/l)] (see **Figure 1**).

At the beginning of the experiment (M_0_), the participants were asked to rate their perceived stress (SUD). After a baseline measurement of approximately 5 min, during which the cardiological parameters were continuously assessed, the first sham-intervention took place (I_1_). Here, the physician administered 2 ml of 0.9% physiological saline solution (NaCl) intravenously together with a standardized verbal neutral suggestion that the intravenously administered solution would not cause any bodily changes “similar to taking a sip of water.” Thereafter, the first post-intervention measurement of physiological parameters was performed (approximately 5 min). At the end, patients were asked again to rate their level of distress on an 11-point numeric rating scale (from 0 = no stress to 10 = maximal stress) and blood samples were taken for a second time (M_2_). Subsequently, the same procedure was performed for the placebo and the nocebo interventions: after a pre-intervention measurement of physiological parameters of approximately 5 min patients were asked to rate perceived stress levels (SUD) (M_3_). Next, 2 ml NaCl was administered intravenously accompanied by a standardized verbal positive suggestion that the intervention would “strengthen the heart,” “blood pressure and heart rate would decrease,” and “breathing would become easier” as the body would be “better supplied with oxygen” (I_3_). Then another post-intervention measuring period (approximately 5 min) was obtained with continuous measurement of physiological parameters. At the end of this period, distress levels were assessed and blood samples were taken (M_4_). Again after a pre-intervention period of approximately 5 min, stress ratings (SUD) were assessed again (M_5_). Finally, the last 2 ml NaCl was administered analogously to the previous conditions, with the verbal suggestion that this intervention would “burden” the heart, it would need to work “stronger and faster,” and “hot flashes” could occur (I_5_). Conclusively, the last post-intervention period (approximately 5 min) was performed with continuous measurement of physiological parameters and assessment of distress levels, and the last blood sample was taken (M_6_). At the end of the examination the study rationale was disclosed to the participants and they were informed about the placebo character of the study with the administered substance being only “water.” Additionally, the individual echocardiography results were reviewed together with the patient.

### Statistical Analysis

Analyses were performed by means of IBM SPSS Statistics 25 with a *p*-value ≤ 0.05 considered as significant. Mean values of HR, SBP, and DBP were calculated for the period from 200 to 20 s prior to the interventions (pre values) and 20 to 200 s after the interventions (post values). Data that did not fit normal distribution were logarithmized. Pre-post changes of HR, SBP, and DBP induced by the neutral, positive, and negative interventions were compared between groups by means of a mixed-design ANOVA with the within-subject factors “time” (pre and post intervention) and “condition” (neutral, positive, and negative), and the between-subject factor “group” (TTC, controls). Subsequently Bonferroni-corrected *post hoc* tests were performed. Due to the absence of a normal distribution, SUD levels were evaluated by using Bonferroni-corrected Wilcoxon signed-rank tests and Kruskal–Wallis tests, respectively; changes of cortisol as well as copeptin levels were calculated using Wilcoxon signed-rank tests, Mann–Whitney–U, and Friedman tests.

## Results

### Baseline Characteristics

TTC patients and controls were comparable with regard to age, employment situation, living situation, and quality of life. The time point of evaluation did not differ between groups and the mean time span between the TTC diagnosis and the examination was 24.61 months (±22.8) (**Table 1**).

### Subjective Units of Distress (SUD)

**Figure 2:**
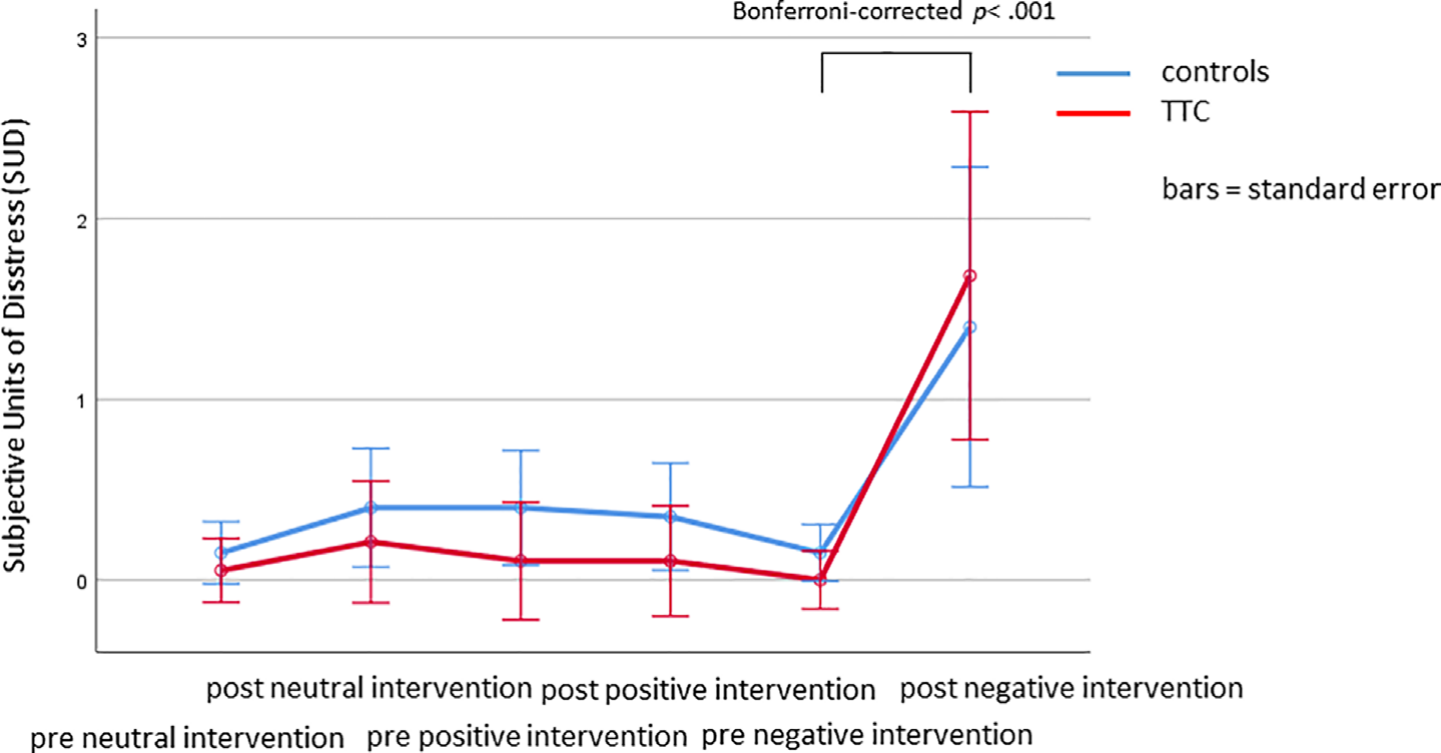
Subjective Units of Distress (means ± SD) before and after the neutral, positive, and negative interventions.

SUD changes from before to after the neutral, positive, and negative intervention were evaluated by using the Wilcoxon signed-rank tests. No significant changes were observed in response to the neutral and positive verbal suggestions (Bonferroni-corrected *p* = .1 and *p* = .06, respectively). However, SUD ratings increased in response to the negative verbal suggestion (Bonferroni-corrected *p* < .001), indicating a nocebo effect on perceived stress. SUD did not differ between patients with a history of TTC and heart-healthy controls at any time point during the experiment (Mann–Whitney–U test, all Bonferroni-corrected *p* > .05) (**Figure 2** and **Table 2**).

**Table 2:** Subjective Units of Distress (SUD), systolic blood pressure (mmHg).

Time point	TTC		Controls	
**Subjective Units of Distress (SUD)**	**Mean**	**SD**	**Mean**	**SD**
Pre neutral suggestion	.15	.49	.05	.22
Post neutral suggestion	.4	.88	.2	.52
Pre positive suggestion	.4	.88	.1	.45
Post positive suggestion	.35	.81	.1	.45
Pre negative suggestion	.15	.49	.0	.0
Post negative suggestion	1.4	1.1	1.6	1.1
**Systolic blood pressure (mmHg)**				
Pre neutral suggestion	120.37	10.86	127.01	18.42
Post neutral suggestion	118.62	8.61	123.11	19.01
Pre positive suggestion	120.45	17.1	130.63	26.47
Post positive suggestion	124.87	13.8	131.53	56.53
Pre negative suggestion	127.72	13.93	125.13	20.42
Post negative suggestion	134.82	22.36	129.1	21.03
Pre neutral suggestion	120.37	10.86	127.01	18.42
**Diastolic blood pressure (mmHg)**				
Pre neutral suggestion	57.17	13.71	61.6	11
Post neutral suggestion	58.0	11.83	59.16	11.1
Pre positive suggestion	53.68	8.6	60.04	13.45
Post positive suggestion	55.62	7.29	59.67	13.41
Pre negative suggestion	57.91	10.41	59.56	13.21
Post negative suggestion	59.1	10.69	61.39	13.23
**Heart rate (bpm)**				
Pre neutral suggestion	54.38	5.46	55.34	7.44
Post neutral suggestion	55.74	5.61	56.95	7.76
Pre positive suggestion	57.92	9.05	56.17	8.32
Post positive suggestion	58.69	8.47	57.57	8.63
Pre negative suggestion	58.21	9.29	57.69	8.73
Post negative suggestion	58.53	8.86	57.63	8.48
**Cortisol (µg/dl)**				
Pre neutral suggestion	13.23	5.46	14.05	7.44
Post neutral suggestion	12.87	5.61	13.54	7.76
Post positive suggestion	12.37	8.47	13.08	8.63
Post negative suggestion	11.99	8.86	12.81	8.48
**Copeptin (pmol/l)**				
Pre neutral suggestion	54.38	20.82	55.34	20.73
Post neutral suggestion	55.74	20.83	56.95	20.65
Post positive suggestion	58.69	20.92	57.57	20.66
Post negative suggestion	58.53	20.80	57.63	20.75

### Systolic Blood Pressure (SBP)

**Figure 3:**
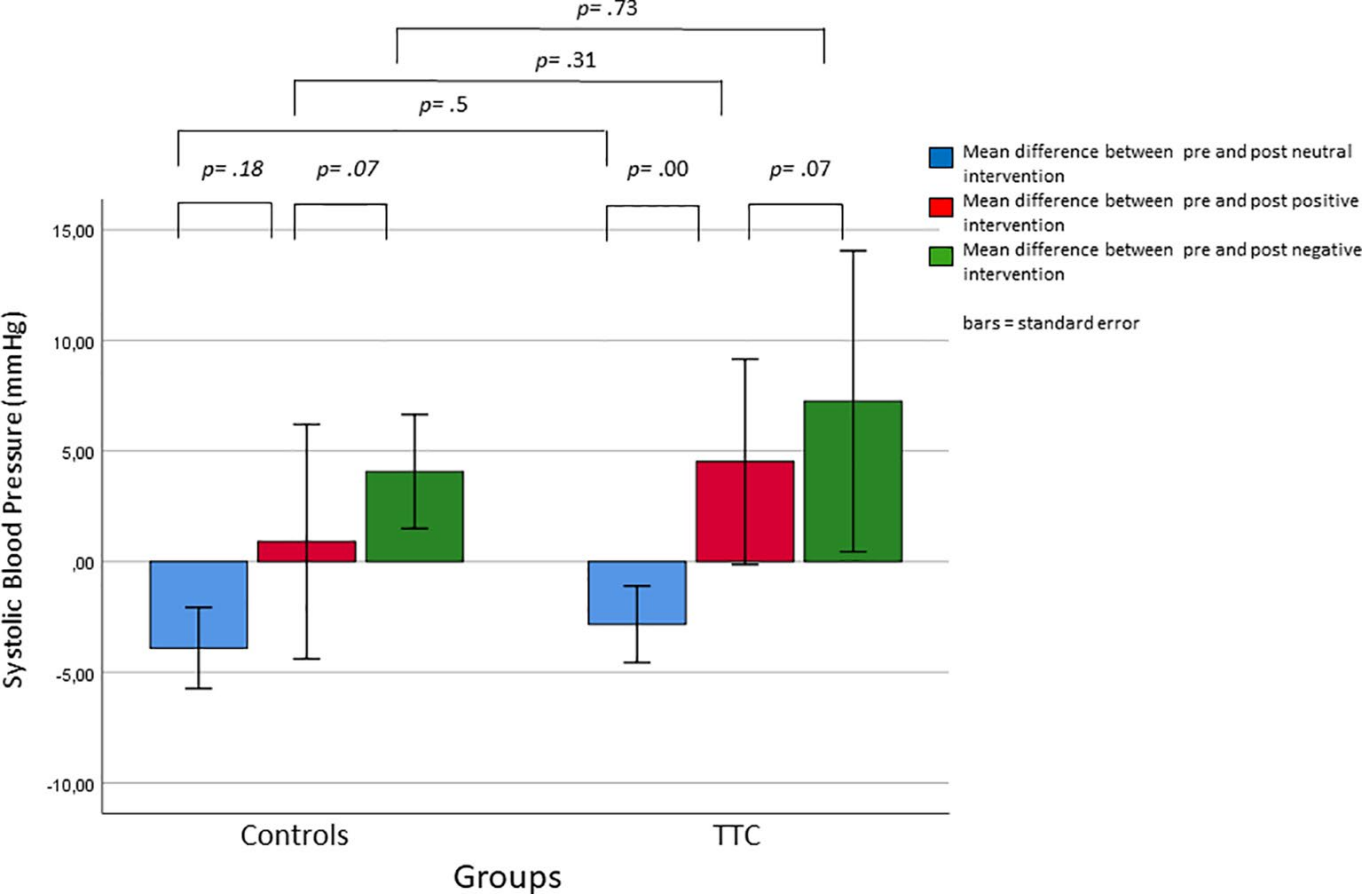
Changes in systolic blood pressure in response to the neutral, positive, and negative placebo interventions.

The mixed-design ANOVA with the within-subject factors “time” (pre, post intervention) and “condition” (neutral, positive, negative) and the between-subject factor “group” (TTC, controls) was used to examine SBP levels. A significant interaction between “time” and “condition” was found (*F*_(2,76)_ = 14.09; *p* < .001). *Post hoc* tests showed higher SBP levels in response to the negative and the positive verbal suggestions as compared to the neutral verbal suggestion (Bonferroni-corrected *p*-values, *p* = .045 and *p* = .002, respectively). There was also a significant main effect for “condition” (*F*_(2,76)_ = 3.2, *p* = .047). Bonferroni-corrected *post hoc* tests, however, revealed no significant difference between conditions. No other main or interaction effects were significant (**Figure 3** and **Table 2**).

### Diastolic Blood Pressure (DBP)

**Figure 4:**
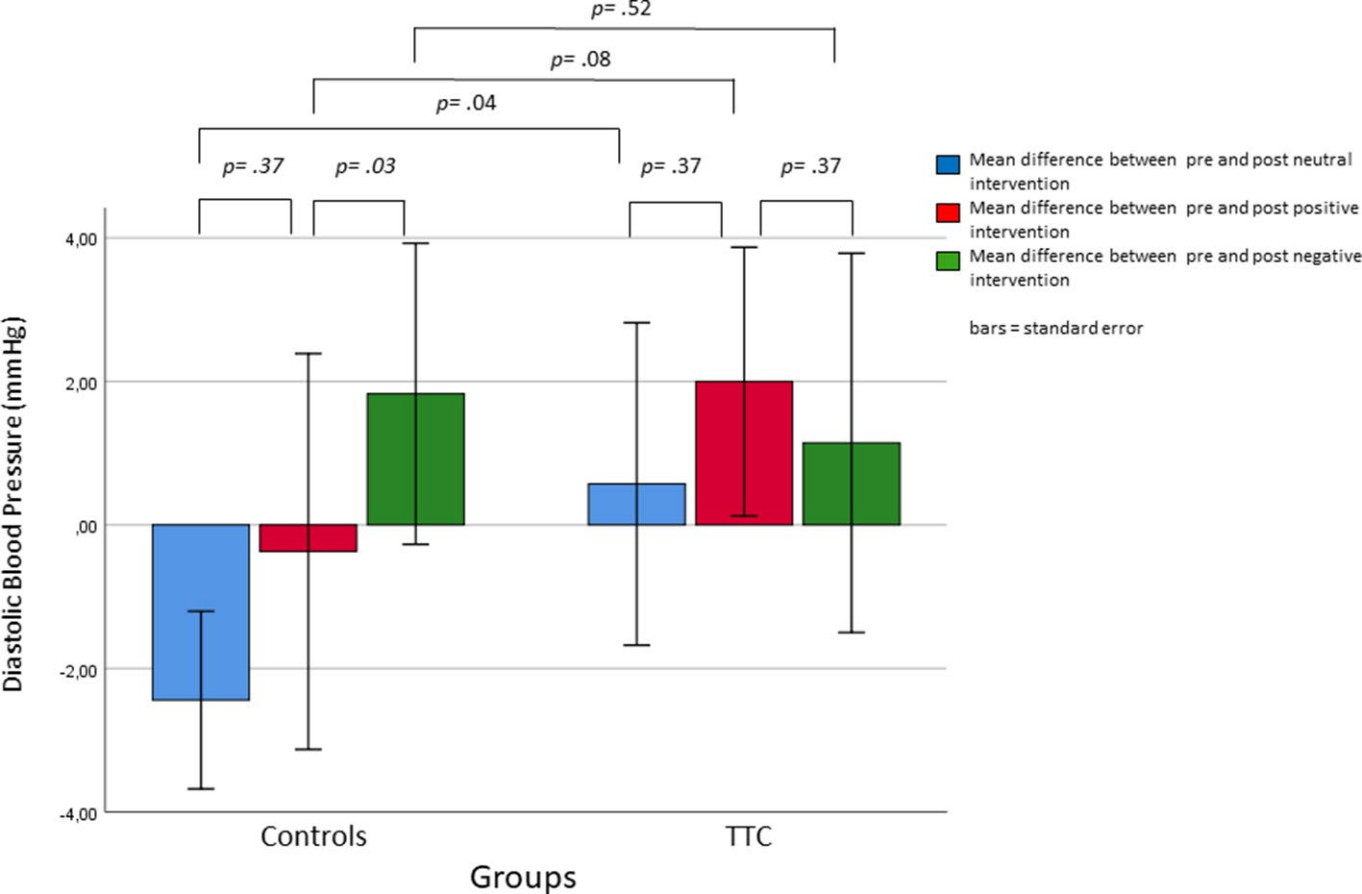
Changes diastolic blood pressure in response to the neutral, positive, and negative placebo interventions.

The mixed-design ANOVA for DBP levels with the within-subject factors “time” (pre andf post intervention) and “condition” (neutral, positive, and negative) and the between-subject factor “group” (TTC and controls) revealed no significant main or interaction effects (**Figure 4** and **Table 2**).

### Heart Rate (HR)

**Figure 5:**
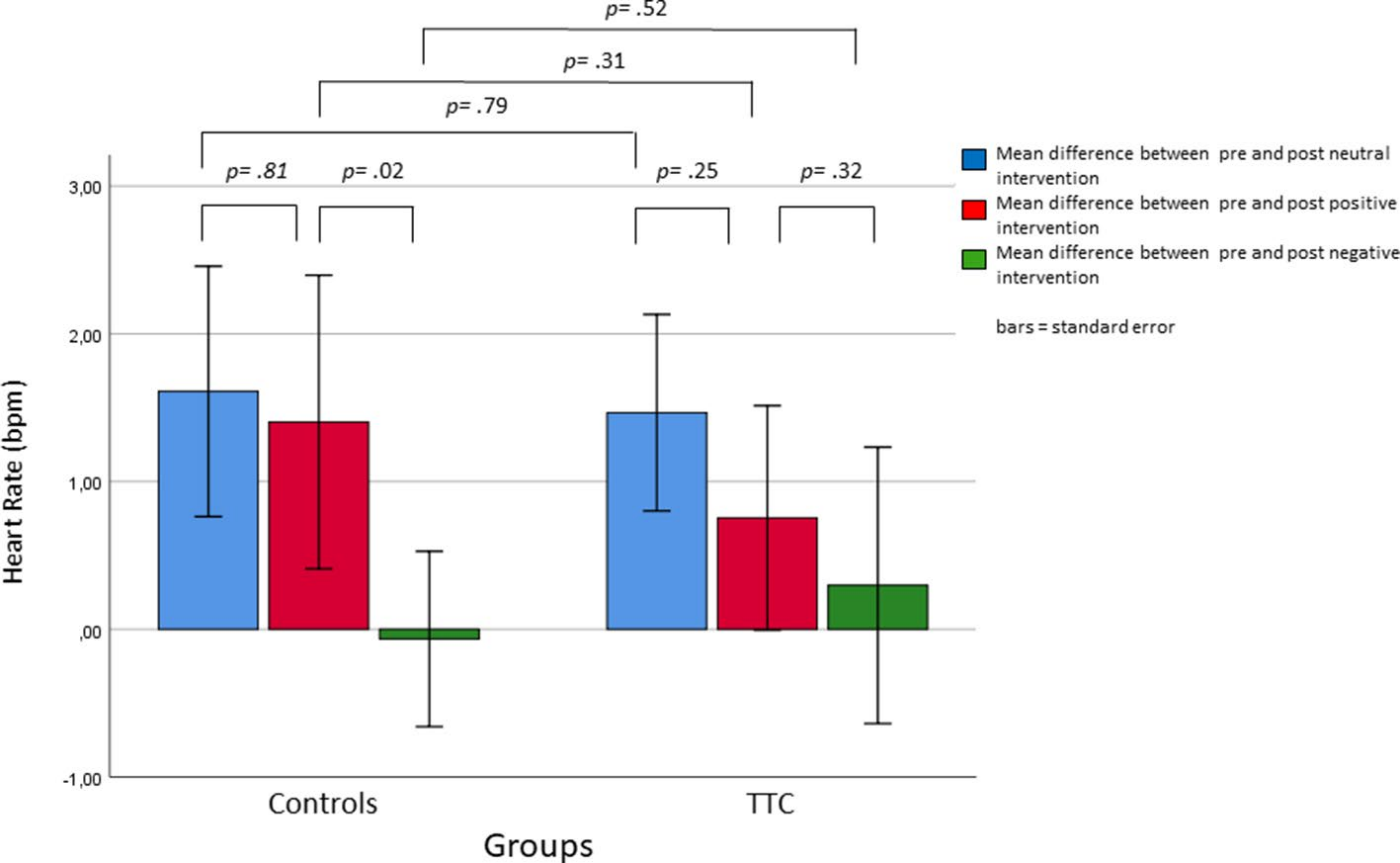
Changes in heart rate in response to the neutral, positive, and negative placebo interventions.

**Figure 6:**
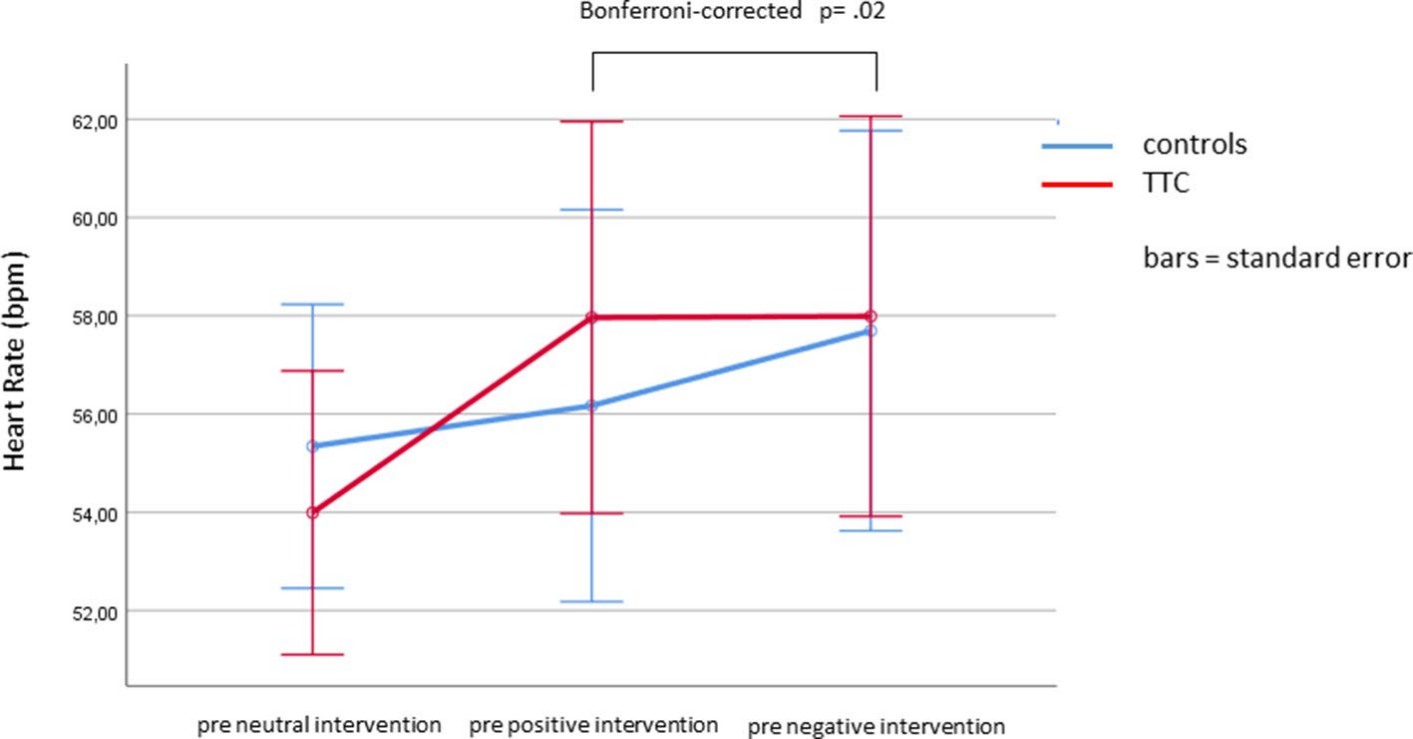
Heart rate (bpm) during the anticipation phases (pre values) of the three interventions.

The mixed-design ANOVA with the within-subject factors “time” (pre and post intervention) and “condition” (neutral, positive, and negative) and the between-subject factor “group” (TTC and controls) for HR levels revealed a significant interaction effect between “time” and “condition” (*F*_(2,76)_ = 5.5; *p* = .01). Simple effects analyses showed that this interaction was due to higher HR levels before the negative verbal suggestion compared to before the positive verbal suggestion, indicating an anticipatory increase of HR (Bonferroni-corrected *p* = .02). Furthermore, a significant main effect of “condition” was found (*F*_(2,78)_ = 5.11, *p* = .01), with higher HR levels in the nocebo condition compared to the neutral condition (Bonferroni-corrected *p* = .037). Finally, the main effect of “time” was significant (*F*_(1,39)_ = 46.8, *p* < .001), which was due to increasing HR levels from before to after the intervention (estimated means ± SE, before: 56.5 ± 1.2 and after: 57.4 ± 1.2). No other main or interaction effects were significant (**Figures 5**, **6** and **Table 2**).

### Humoral Stress Markers

**Figure 7:**
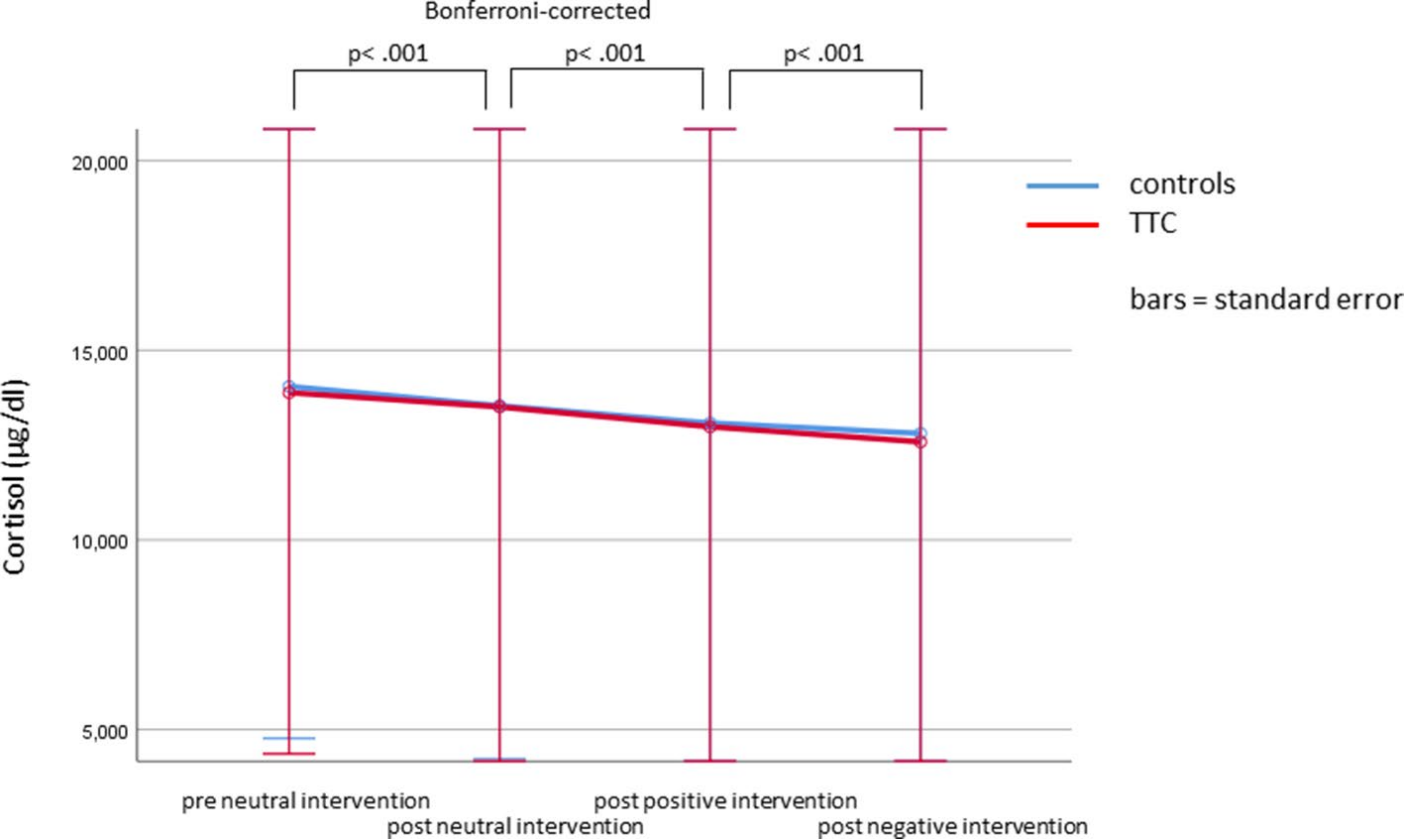
Plasma cortisol levels according to interventions.

Cortisol levels at baseline and after the neutral, the positive and the negative verbal suggestions were compared by Friedman tests. Results revealed a significant difference between conditions (*x* ^2^ = 64.3, *p* < .001), which was due to a significant decrease of cortisol levels from condition to condition (Wilcoxon tests, all Bonferroni-corrected *p* < .001). In no condition significant group differences between TTC patients and controls were observed (Mann–Whitney–U test, all Bonferroni-corrected *p*-values = 1) (**Table 2**). A Friedman test for copeptin levels at baseline and after the neutral, the positive and the negative verbal suggestions revealed no significant differences between conditions (*p* = .84). In no condition significant differences between TTC patients and controls were observed (Mann–Whitney–U test, all Bonferroni-corrected *p* = 1) (**Figure 7** and **Table 2**).

## Discussion

In this study, we investigated cardiac, psychological, and endocrine stress responses to placebo and nocebo interventions targeting the heart in patients with a history of TTC and matched heart-healthy controls. Although the pathophysiology underlying TTC is not yet entirely clear, a dysfunctional, overmodulated stress response with enhanced sympathetic stimulation might be of key importance (19). We expected that physiological and behavioral responses to placebo and nocebo interventions would be more pronounced in patients with a history of TTC compared to controls than in heart-healthy controls. In our study a significant nocebo effect on subjective units of distress was detected for the whole group of 40 participants. Furthermore, HR increased significantly before the nocebo intervention, possibly indicating anticipatory anxiety towards the upcoming negative intervention. In addition, SBP levels increased significantly in response to both, the placebo and nocebo interventions, suggesting a possible nocebo effect on SBP. Significant alterations of DBP, cortisol and copeptin due to the interventions failed to appear. Contrary to our expectations, none of these responses differed between TTC patients and heart-healthy controls.

Evidence regarding placebo effects on end organ functions regulated by the ANS (e.g., cardiovascular or gastric functions) is less clear compared to the accumulating evidence for placebo effects on functions associated with the central nervous system [e.g., pain and itch, e.g. Refs. (44–46)]. The ANS is characterized by high functional specificity provided through elaborated afferent and efferent fibers. Hence, it is not surprising that placebo and nocebo interventions targeting end-organ functions controlled by the ANS can display a high target-specificity (10, 47). The present study adds to this field of placebo research in addressing cardiac parameters that are under control of the autonomic nervous system (HR, SPB, and DBP), as well as subjective stress ratings (SUD) and humoral correlates (copeptin and cortisol). To our knowledge this is one of the first experimental studies, and the first placebo study, in patients with a history of TTC.

Our observations of significant effects from placebo and nocebo interventions on SPB and HR but not on DBP are in accordance with previous studies, which investigated placebo and nocebo effects on cardiovascular parameters by means of verbal suggestions (13, 48). Former investigations that aimed to induce BP changes in healthy individuals by means of a placebo-spray in combination with verbal suggestions for instance, assumed that the absence of significant BP alterations could potentially be explained by lacking associations between memories of physiological or mental states with specific autonomic changes in the brain, which might be a necessary condition for verbal suggestions to induce the intended effects (49). This explanation was linked to the central organizational principle of the brain named, the “reuse of neural circuity,” supposing that neural circuits established for a specific purpose, diversify or exploit to new uses, without losing their genuine function (50). This explanatory approach might also give insightful hints for the results of our study. A link between memories of BD and HR decreases and specific autonomic changes in the brain that could be crucial for the targeted physiological changes might not have been available.

Also the disclosure of the fixed order of the interventions, with the negative intervention being at the end, might have prevented the positive verbal suggestions to evoke HR and BP decrease. The increase of HR prior to the beginning of the nocebo intervention might be linked to the disclosure of the chronological order of interventions as well and could indicate anticipatory anxiety towards the nocebo intervention. Lyby and colleagues could show that fear can eliminate placebo effects induced by verbal suggestions (51). In this regard several imaging studies especially from the area of pain indicate that there is altered activity in the cortical nociceptor network already during the anticipation of pain (52, 53). Moreover, the perception of pain is not exclusively depending on the specific noxious stimulus. Attention, expectation and reappraisal seem to play an important role in the cognitive modulation of pain (54). Among other brain regions [e.g., dorsolateral prefrontal cortex (DLPFC) or the periaqueductal gray (PAG)], especially the rostral anterior cingulate cortex (rACC) seems to play an important role in the nociceptive network and reveals complex response patterns provoked by placebo interventions, but also during anticipation phases (55–59). An activation likelihood estimation meta-analysis also underlines the impact of negative expectations resulting from past experiences and present information on pain perception, which in turn might lead to higher pain intensity (60). Therefore, the anticipation of the negative intervention might explain the absence of relaxing effects due to the positive verbal suggestion and the increase of HR prior to the negative verbal suggestion. Nocebo effects (especially in the area of pain) have proven to be associated with complex biochemical and neuroendocrine mechanisms that seem to be connected to anticipatory anxiety (44). This suggests the activation of the HPA or SNS, which build the main peripheral pathways of the human stress system. The HPA axis regulates the release of cortisol that has been shown to be proportionate to the degree of stress on a peripheral level. In our study cortisol levels did not change as a response to the interventions, as it could be seen in previous studies on nocebo hyperalgesia but “naturally” decreased during the examination (61, 62). A similar phenomenon could be seen in a study done by Meissner et al. who examined the predictive value of cortisol on motion sickness (63) or Benedetti et al. who showed that placebo and nocebo effects in cortisol secretion could not be induced by verbal suggestions, but were affected by pharmacological conditioning (3). A meta-analysis, again in the area of pain, showed that the combination of verbal suggestions and conditioning induces larger placebo and nocebo effects than verbal suggestions alone (64, 65). Colloca and colleagues concluded that conditioning is less important in nocebo hyperalgesia compared to placebo analgesia (1). Unintended expectations and stimulus pairings could have been developed through the TTC patient’s experiences during their disease history that might have led to a “blending” of expectation- and conditioning-induced effects in our examination (66).

The question of whether TTC is a transient, reversible disease, or is based on an enduring pathology affecting the sympathetic nervous system, is not yet fully clarified. It is widely believed that the suspected, exaggerated sympathetic activation within the acute phase of TCC is triggered by a precedent, mostly unexpected stressful life event [e.g., Ref. (21)]. The assumption that the normalization of the shape of the left ventricle and the systolic LVEF is accompanied by a regulation of the underlying sympathetic activation, would in turn explain the lacking difference between TTCs and heart-healthy controls. Additionally, recent studies indicate that the exposure to repeated stressors (in contrast to a single life event) is associated with the onset of TTC (67, 68), the authors argued that long-term stressful conditions might have led to an increased vulnerability towards strong emotional or physical stressors triggering the development of TTC. Within our study, positive as well as negative interventions were announced far in advance, took place in the “save environment” of the hospital and might therefore not have served as suitable stimuli for an exaggerated activation of the sympathetic nervous system. Another recent study focused on altered β-adrenergic signaling in TTC cardiomyocytes derived from pluripotent stem cells to explore whether genetic susceptibility underlies the pathophysiology of TTC. These findings point at a complex, multifactorial etiology of TTC with genetic predispositions combined with environmental factors such as age, postmenopausal hormonal status and stressful life events (69). At the cellular level, Borchert and colleagues could demonstrate that TTC phenotype was associated with enhanced β-adrenergic signaling and higher sensitivity to catecholamine-induced toxicity (70). These considerations might be further promising regarding distinguishing features between TTC and heart healthy individuals.

Although the sample size of 20 TTC patients is comparably high considering the prevalence of 0.07–2.3% of patients suspected with an ACS, a larger number of participants in our study would have been desirable. As a further issue the participants’ medication intake (e.g., β-blocker) needs to be considered. Although the intake of antihypertensive medication was relatively similar in both groups, this could have led to a dampening effect of sympathetic activation and might therefore have reduced differences between groups. Furthermore, in the light of the explanations above, a combining of classical conditioning and verbal suggestions might have improved especially the placebo response but also the nocebo response. It could have shed new light on the impact of conditioning and verbal suggestions (resp. explicit expectations) on placebo and nocebo effects within the autonomic nervous system. A further limitation might be the variety of time spans between the cardiac event and the investigation that is attributed to the low prevalence of TTC. If we would have included patients within their acute phase only, the recruitment period would have been enormously long, which would have meant that constancy in further parameters, for instance examiner or examination rooms, could not have been guaranteed. If TTC is seen as a reversible disease or a maintaining pathology in stress processing, a predefinition of one or more specific time points (e.g., within the acute phase together with a two-year follow-up) needs to be considered in a further study. Due to standardization resp. generalization reasons (especially considering the relatively small sample size) the chronological order of the three interventions was standardized. Future studies should consider a cross-over design with a randomized order. The observation that the positive verbal suggestion did not reduce perceived stress is most probably due to a floor effect, since stress at baseline was very low (see **Figure 2**). Finally, the consideration that anticipatory anxiety might have prevented the induction of a placebo effect suggests to additionally collect fear ratings during the course of the intervention.

Summarizing, this study was the first to investigate effects of positive and negative verbal suggestions in combination with the intravenous application of saline solution on cardiac parameters in patients with a history of TTC compared to controls. Only an increase of SBP could be observed as a response to both positive and negative suggestions. Secondly the increase of SBP as a response to the nocebo intervention was congruently accompanied by higher levels of SUD. The increase of HR prior to the beginning of the nocebo intervention is possibly associated to anticipatory anxiety of the nocebo intervention. Our hypothesis that the cardiac response towards placebo and nocebo interventions in patients with a history of TTC would be different from those of heart-healthy controls could not be confirmed with our data, a TTC, on average diagnosed two years ago, does not appear to have an influence on the responsivity to placebo resp. nocebo interventions. This becomes even more important considering the fact that the etiology of TTC is not yet fully explained. The assumption that an altered sympathetic disposition might build the precondition for the pathophysiological cascade of TTC-patient’s myocardium within the acute phase, could not be verified with our placebo resp. nocebo interventions, at least at the time of our examination, on average, two years after the acute phase.

## Ethics Statement

This study was carried out in accordance with the recommendations of the Code of Conduct of the Technical University Munich, Germany, with written informed consent from all subjects. All subjects gave written informed consent in accordance with the Declaration of Helsinki. The protocol was approved by the institutional board of the Technical University Munich, Germany.

## Author Contributions

EO, KM, SS, GS, DS, AM, CB, HE, and JR designed the experiment. EO, SS, and DS performed the experiment. EO, KM, AM, DS, SB, K-HL and SH analyzed the data. EO drafted the first version of the manuscript. All authors interpreted the data and critically reviewed the manuscript.

## Funding

The study was supported by the Deutsches Herzzentrum, Technische Universitaet, Munich, Germany. This work was supported by the German Research Foundation (DFG) and the Technical University of Munich within the funding program Open Access Publishing.

## Conflict of Interest Statement

The authors declare that the research was conducted in the absence of any commercial or financial relationships that could be construed as a potential conflict of interest.

## References

[1] Colloca L Sigaudo M Benedetti F The role of learning in nocebo and placebo effects. Pain (2008) 136(1–2):211–8. 10.1016/j.pain.2008.02.006 18372113

[2] De Houwer J A functional-cognitive perspective on the relation between conditioning and placebo research. Int Rev Neurobiol (2018) 138:95–111. 10.1016/bs.irn.2018.01.007 29681337

[3] Benedetti F Pollo A Lopiano L Lanotte M Vighetti S Rainero I Conscious expectation and unconscious conditioning in analgesic, motor, and hormonal placebo/nocebo responses. J Neurosci (2003) 23(10):4315–23. 10.1523/JNEUROSCI.23-10-04315.2003 PMC6741114 12764120

[4] Benedetti F Placebo effects, 2nd ed New York, NY, US: Oxford University Press (2014). 10.1093/acprof:oso/9780198705086.001.0001

[5] Aslaksen PM Bystad M Vambheim SM Flaten MA Gender differences in placebo analgesia: event-related potentials and emotional modulation. Psychosom Med (2011) 73(2):193–9. 10.1097/PSY.0b013e3182080d73 21217098

[6] Colloca L Klinger R Flor H Bingel U Placebo analgesia: psychological and neurobiological mechanisms. Pain (2013) 154(4):511–4. 10.1016/j.pain.2013.02.002 PMC3626115 23473783

[7] Colloca L Benedetti F Nocebo hyperalgesia: how anxiety is turned into pain. Curr Opin Anesthesiology (2007) 20(5):435–9. 10.1097/ACO.0b013e3282b972fb 17873596

[8] de la Fuente-Fernandez R Ruth TJ Sossi V Schulzer M Calne DB Stoessl AJ Expectation and dopamine release: mechanism of the placebo effect in Parkinson’s disease. Science (2001) 293(5532):1164–6. 10.1126/science.1060937 11498597

[9] Benedetti F Arduino C Amanzio M Somatotopic activation of opioid systems by target-directed expectations of analgesia. J Neurosci (1999) 19(9):3639–48. 10.1523/JNEUROSCI.19-09-03639.1999 PMC6782226 10212322

[10] Meissner K The placebo effect and the autonomic nervous system: evidence for an intimate relationship. Philos Trans Royal Society B Biol Sci (2011) 366(1572):1808–17. 10.1098/rstb.2010.0403 PMC3130407 21576138

[11] Meissner K Fässler M Rücker G Kleijnen J Hróbjartsson A Schneider A Differential effectiveness of placebo treatments: a systematic review of migraine prophylaxis. JAMA Internal Med (2013) 173(21):1941–51. 10.1001/jamainternmed.2013.10391 24126676

[12] Ronel J Mehilli J Ladwig K-H Blättler H Oversohl N Byrne RA Effects of verbal suggestion on coronary arteries: results of a randomized controlled experimental investigation during coronary angiography. Am Heart J (2011) 162(3):507–11. 10.1016/j.ahj.2011.06.016 21884868

[13] Amigo I Cuesta V Fernández A González A The effect of verbal instructions on blood pressure measurement. J Hypertens (1993) 11(3):293–6. 10.1097/00004872-199303000-00010 8387087

[14] El Mahmoud R Mansencal N Pilliere R Leyer F Abbou N Michaud P Prevalence and characteristics of left ventricular outflow tract obstruction in Tako-Tsubo syndrome. Am Heart J (2008) 156(3):543–8. 10.1016/j.ahj.2008.05.002 18760139

[15] Maron BJ Towbin JA Thiene G Antzelevitch C Corrado D Arnett D Contemporary definitions and classification of the cardiomyopathies: an American heart association scientific statement from the council on clinical cardiology, heart failure and transplantation committee; quality of care and outcomes research and functional genomics and translational biology interdisciplinary working groups; and council on epidemiology and prevention. Circulation (2006) 113(14):1807–16. 10.1161/CIRCULATIONAHA.106.174287 16567565

[16] Kurisu S Sato H Kawagoe T Ishihara M Shimatani Y Nishioka K Tako-tsubo-like left ventricular dysfunction with ST-segment elevation: a novel cardiac syndrome mimicking acute myocardial infarction. Am Heart J (2002) 143(3):448–55. 10.1067/mhj.2002.120403 11868050

[17] Haghi D Roehm S Hamm K Harder N Suselbeck T Borggrefe M Takotsubo cardiomyopathy is not due to plaque rupture: an intravascular ultrasound study. Clin Cardiol (2010) 33(5):307–10. 10.1002/clc.20747 PMC6653111 20513070

[18] Medeiros K O’Connor MJ Baicu CF Fitzgibbons TP Shaw P Tighe DA Systolic and diastolic mechanics in stress cardiomyopathy CLINICAL PERSPECTIVE. Circulation (2014) 129(16):1659. 24503950 10.1161/CIRCULATIONAHA.113.002781

[19] Wittstein IS The sympathetic nervous system in the pathogenesis of takotsubo syndrome. Heart Failure Clinics (2016) 12(4):485–98. 10.1016/j.hfc.2016.06.012 27638019

[20] Deshmukh A Kumar G Pant S Rihal C Murugiah K Mehta JL Prevalence of Takotsubo cardiomyopathy in the United States. Am Heart J (2012) 164(1):66–71 e1. 10.1016/j.ahj.2012.03.020 22795284

[21] Gianni M Dentali F Grandi AM Sumner G Hiralal R Lonn E Apical ballooning syndrome or takotsubo cardiomyopathy: a systematic review. Eur Heart J (2006) 27(13):1523–9. 10.1093/eurheartj/ehl032 16720686

[22] Klinceva M Widimsky P Pesl L Stasek J Tousek F Vambera M Prevalence of stress-induced myocardial stunning (Tako-Tsubo cardiomyopathy) among patients undergoing emergency coronary angiography for suspected acute myocardial infarction. Int J Cardiol (2007) 120(3):411–3. 10.1016/j.ijcard.2006.07.228 17098304

[23] Kurowski V Kaiser A von Hof K Killermann DP Mayer B Hartmann F Apical and midventricular transient left ventricular dysfunction syndrome (tako-tsubo cardiomyopathy): frequency, mechanisms, and prognosis. Chest (2007) 132(3):809–16. 10.1378/chest.07-0608 17573507

[24] Parodi G Del Pace S Carrabba N Salvadori C Memisha G Simonetti I Incidence, clinical findings, and outcome of women with left ventricular apical ballooning syndrome. Am J Cardiol (2007) 99(2):182–5. 10.1016/j.amjcard.2006.07.080 17223415

[25] Templin C Ghadri JR Diekmann J Napp LC Bataiosu DR Jaguszewski M Clinical features and outcomes of takotsubo (stress) cardiomyopathy. N Engl J Med (2015) 373(10):929–38. 10.1056/NEJMoa1406761 26332547

[26] Ghadri JR Sarcon A Diekmann J Bataiosu DR Cammann VL Jurisic S Happy heart syndrome: role of positive emotional stress in takotsubo syndrome. Eur Heart J (2016) 37(37):2823–9. 10.1093/eurheartj/ehv757 PMC5841222 26935270

[27] Katsanos S Filippatou A Ruschitzka F Filippatos G Positive emotions and Takotsubo syndrome:’happy heart’or ‘Diagoras’ syndrome? Eur Heart J (2016) 37(37):2821–2. 10.1093/eurheartj/ehw197 27252450

[28] Sharkey SW Windenburg DC Lesser JR Maron MS Hauser RG Lesser JN Natural history and expansive clinical profile of stress (tako-tsubo) cardiomyopathy. J Am Coll Cardiol (2010) 55(4):333–41. 10.1016/j.jacc.2009.08.057 20117439

[29] Elesber AA Prasad A Lennon RJ Wright RS Lerman A Rihal CS Four-year recurrence rate and prognosis of the apical ballooning syndrome. J Am Coll Cardiol (2007) 50(5):448–52. 10.1016/j.jacc.2007.03.050 17662398

[30] Ghadri J-R Wittstein IS Prasad A Sharkey S Dote K Akashi YJ International expert consensus document on takotsubo syndrome (part I): clinical characteristics, diagnostic criteria, and pathophysiology. Eur Heart J (2018) 39(22):2032–46. 10.1093/eurheartj/ehy076 PMC5991216 29850871

[31] Redfors B Vedad R Angeras O Ramunddal T Petursson P Haraldsson I Mortality in takotsubo syndrome is similar to mortality in myocardial infarction - A report from the SWEDEHEART registry. Int J Cardiol (2015) 185:282–9. 10.1016/j.ijcard.2015.03.162 25818540

[32] Sobue Y Watanabe E Ichikawa T Koshikawa M Yamamoto M Harada M Physically triggered Takotsubo cardiomyopathy has a higher in-hospital mortality rate. Int J Cardiol (2017) 235:87–93. 10.1016/j.ijcard.2017.02.090 28262347

[33] Burgdorf C Kurowski V Bonnemeier H Schunkert H Radke PW Long-term prognosis of the transient left ventricular dysfunction syndrome (Tako-Tsubo cardiomyopathy): focus on malignancies. Eur J Heart Fail (2008) 10(10):1015–9. 10.1016/j.ejheart.2008.07.008 18692439

[34] Burgdorf C Nef HM Haghi D Kurowski V Radke PW Tako-tsubo (stress-induced) cardiomyopathy and cancer. Ann Intern Med (2010) 152(12):830–1. 10.7326/0003-4819-152-12-201006150-00026 20547921

[35] Singh K Carson K Shah R Sawhney G Singh B Parsaik A Meta-analysis of clinical correlates of acute mortality in takotsubo cardiomyopathy. Am J Cardiol (2014) 113(8):1420–8. 10.1016/j.amjcard.2014.01.419 24685327

[36] Singh K Carson K Usmani Z Sawhney G Shah R Horowitz J Systematic review and meta-analysis of incidence and correlates of recurrence of takotsubo cardiomyopathy. Int J Cardiol (2014) 174(3):696–701. 10.1016/j.ijcard.2014.04.221 24809923

[37] Madhavan M Prasad A Proposed Mayo Clinic criteria for the diagnosis of Tako-Tsubo cardiomyopathy and long-term prognosis. Herz (2010) 35(4):240–3. 10.1007/s00059-010-3339-x 20582391

[38] Kresh JY Izrailtyan I Evolution in functional complexity of heart rate dynamics: a measure of cardiac allograft adaptability. Am J Physiol (1998) 275(3 Pt 2):R720–7. 10.1152/ajpregu.1998.275.3.R720 9728068

[39] Neumeister B Böhm BO Adler S Klinikleitfaden Labordiagnostik. Elsevier Health Sciences (2018).

[40] Katan M Morgenthaler N Widmer I Puder JJ Konig C Muller B Copeptin, a stable peptide derived from the vasopressin precursor, correlates with the individual stress level. Neuroendocrinology Letters (2008) 29(3):341–6. 18580851

[41] Struck J Morgenthaler NG Bergmann A Copeptin, a stable peptide derived from the vasopressin precursor, is elevated in serum of sepsis patients. Peptides (2005) 26(12):2500–4. 10.1016/j.peptides.2005.04.019 15922490

[42] Widmer IE Puder JJ Ko¨nig C Pargger H Zerkowski HR Girard Jr Cortisol response in relation to the severity of stress and illness. J Clin Endocr Metabolism (2005) 90(8):4579–86. 10.1210/jc.2005-0354 15886236

[43] Grelotti DJ Kaptchuk TJ Placebo by proxy. BMJ (Clinical research ed.) (2011) 343, d4345. 10.1136/bmj.d4345 PMC3230083 21835868

[44] Benedetti F Lanotte M Lopiano L Colloca L When words are painful: unraveling the mechanisms of the nocebo effect. Neuroscience (2007) 147(2):260–71. 10.1016/j.neuroscience.2007.02.020 17379417

[45] van Laarhoven AI Vogelaar ML Wilder-Smith OH van Riel PL de Kerkhof PC Kraaimaat FW Induction of nocebo and placebo effects on itch and pain by verbal suggestions. PAIN ^®^ (2011) 152(7):1486–94. 10.1016/j.pain.2011.01.043 21353388

[46] van Laarhoven AI van der Sman-Mauriks IM Donders ART Pronk MC de Kerkhof PC Evers AW Placebo effects on itch: a meta-analysis of clinical trials of patients with dermatological conditions. J Invest Dermatol (2015) 135(5):1234–43. 10.1038/jid.2014.522 25609025

[47] Meissner K *Placebo responses on cardiovascular, gastrointestinal, and respiratory organ functions* In:Placebo., Berlin, Heidelberg: Springer (2014). p. 183–203. 10.1007/978-3-662-44519-8_11 25304533

[48] Agras SW Horne M Taylor BC Expectation and the blood-pressure-lowering effects of relaxation. Psychosom Med (1982) 44(4):389–95. 10.1097/00006842-198209000-00006 6755527

[49] Zimmermann-Viehoff F Meissner K Koch J Weber CS Richter S Deter HC Autonomic effects of suggestive placebo interventions to increase or decrease blood pressure: a randomized controlled trial in healthy subjects. J Psychosom Res (2013) 75(1):32–5. 10.1016/j.jpsychores.2013.03.011 23751235

[50] Anderson ML Neural reuse: a fundamental organizational principle of the brain. Behav Brain Sci (2010) 33(4):245–66. 10.1017/S0140525X10000853 20964882

[51] Lyby PS Forsberg JT Åsli O Flaten MA Induced fear reduces the effectiveness of a placebo intervention on pain. PAIN ^®^ (2012) 153(5):1114–21. 10.1016/j.pain.2012.02.042 22464696

[52] Ploghaus A Tracey I Clare S Gati JS Rawlins JNP Matthews PM Learning about pain: the neural substrate of the prediction error for aversive events. Proc Natl Academy Sci (2000) 97(16):9281–6. 10.1073/pnas.160266497 PMC16859 10908676

[53] Porro CA Baraldi P Pagnoni G Serafini M Facchin P Maieron M Does anticipation of pain affect cortical nociceptive systems? J Neurosci (2002) 22(8):3206–14. 10.1523/JNEUROSCI.22-08-03206.2002 PMC6757517 11943821

[54] Wiech K Ploner M Tracey I Neurocognitive aspects of pain perception. Trends Cognit Sci (2008) 12(8):306–13. 10.1016/j.tics.2008.05.005 18606561

[55] Ritter C Hebart MN Wolbers T Bingel U Representation of spatial information in key areas of the descending pain modulatory system. J Neurosci (2014) 34(13):4634–9. 10.1523/JNEUROSCI.4342-13.2014 PMC6608122 24672009

[56] Petrovic P Kalso E Petersson KM Ingvar M Placebo and opioid analgesia– imaging a shared neuronal network. Science (2002) 295(5560):1737–40. 10.1126/science.1067176 11834781

[57] Geuter S Eippert F Attar CH Büchel C Cortical and subcortical responses to high and low effective placebo treatments. Neuroimage (2013) 67:227–36. 10.1016/j.neuroimage.2012.11.029 PMC3578963 23201367

[58] Watson A El-Deredy W Iannetti GD Lloyd D Tracey I Vogt BA Placebo conditioning and placebo analgesia modulate a common brain network during pain anticipation and perception. PAIN ^®^ (2009) 145(1–2): 24–30. 10.1016/j.pain.2009.04.003 19523766 PMC2743811

[59] Wager TD Rilling JK Smith EE Sokolik A Casey KL Davidson RJ Placebo-induced changes in FMRI in the anticipation and experience of pain. Science (2004) 303(5661):1162–7. 10.1126/science.1093065 14976306

[60] Palermo S Benedetti F Costa T Amanzio M Pain anticipation: an activation likelihood estimation meta-analysis of brain imaging studies. Human brain mapping (2015) 36(5):1648–61. 10.1002/hbm.22727 PMC6869158 25529840

[61] Johansen O Brox J Flaten MA Placebo and nocebo responses, cortisol, and circulating beta-endorphin. Psychosom Med (2003) 65(5):786–90. 10.1097/01.PSY.0000082626.56217.CF 14508021

[62] Greenberg G Haraway MM Comparative psychology: a handbook. New York & London:Taylor & Francis (1998). 10.4324/9780203826492

[63] Meissner K Enck P Muth E Kellermann S Klosterhalfen S Cortisol levels predict motion sickness tolerance in women but not in men. Physiol Behav (2009) 97(1):102–6. 10.1016/j.physbeh.2009.02.007 19419665

[64] Petersen GL Finnerup NB Colloca L Amanzio M Price DD Jensen TS The magnitude of nocebo effects in pain: a meta-analysis. PAIN® (2014) 155(8):1426–34. 10.1016/j.pain.2014.04.016 PMC4213146 24780622

[65] Vase L Riley JL II Price DD A comparison of placebo effects in clinical analgesic trials versus studies of placebo analgesia. Pain (2002) 99(3):443–52. 10.1016/S0304-3959(02)00205-1 12406519

[66] Montgomery GH Kirsch I Classical conditioning and the placebo effect. Pain (1997) 72(1–2):107–13. 10.1016/S0304-3959(97)00016-X 9272794

[67] Wallström S Ulin K Määttä S Omerovic E Ekman I Impact of long-term stress in Takotsubo syndrome: Experience of patients. Eur J Cardiovasc Nurs (2016) 15(7):522–8. 10.1177/1474515115618568 PMC5134193 26572162

[68] Rosman L Dunsiger S Salmoirago-Blotcher E Cumulative impact of stressful life events on the development of Takotsubo cardiomyopathy. Ann Behav Med (2017) 51(6):925–30. 10.1007/s12160-017-9908-y 28417437

[69] Lyon A Stress in a dish. Explor Mech Takotsubo Syndrome (2017) 70(8):992–5. 10.1016/j.jacc.2017.07.716 28818209

[70] Borchert T Hubscher D Guessoum CI Lam TD Ghadri JR Schellinger IN Catecholamine-dependent beta-adrenergic signaling in a pluripotent stem cell model of takotsubo cardiomyopathy. J Am Coll Cardiol. (2017) 70(8):975–91. 10.1016/j.jacc.2017.06.061 28818208

